# Osteoblastoma: When the Treatment Is Not Minimally Invasive, an Overview

**DOI:** 10.3390/jcm10204645

**Published:** 2021-10-10

**Authors:** Carmine Zoccali, Mariangela Novello, Francesco Arrigoni, Alessandra Scotto di Uccio, Dario Attala, Virginia Ferraresi

**Affiliations:** 1 Oncological Orthopaedics Department, IRCCS—Regina Elena National Cancer Institute, Via Elio Chianesi 53, 00144 Rome, Italy; 2 Pathology Department, IRCCS—Regina Elena National Cancer Institute, Via Elio Chianesi 53, 00144 Rome, Italy; mariangelanv@gmail.com (M.N.); dario.attala@ifo.gov.it (D.A.); 3 Department of Biotechnological and Applied Clinical Sciences, University of L’Aquila, Via Vetoio 1, 67100 L’Aquila, Italy; arrigoni.francesco@gmail.com; 4 Hepato-Biliary and Organ Transplant Unit, School of General Surgery, Sapienza University, Viale del Policlinico 155, 00161 Rome, Italy; allascotto@gmail.com; 5 Oncology Department, IRCCS—Regina Elena National Cancer Institute, Via Elio Chianesi 53, 00144 Rome, Italy; virginia.ferraresi@ifo.gov.it

**Keywords:** osteoblastoma, radiofrequency thermoablation, cryotherapy, benign bone tumor

## Abstract

Background: osteoblastoma is a bone-forming tumor accounting for about 1% of all primary bone tumors and 3% of benign bone tumors. The gold-standard treatment is surgical excision; nevertheless, minimally invasive radiological techniques such as thermoablation and, more recently, high intensity focused ultrasound are gaining more importance. The aim of the present paper is to analyze surgical indications based on our experience and on the evidences in the literature. Methods: all patients affected by osteoblastoma who underwent surgical excision in January 2009 and December 2018 were reviewed; eleven patients were enrolled in the study. The epidemiological aspects, size of the disease and site of onset, symptoms, surgery type, indications, and results are reported for every case. Results: all treatments were based on a preoperative diagnosis; pain was constant in all cases. Intralesional surgeries were performed in 9 out of 11 cases; the remaining 2 cases underwent wide resection. No early or late complications occurred after the surgical procedure. The indications for surgery were lesions very close to nerves or joints, unclear diagnosis, risk of fracture, lesion too large for radiofrequency thermoablation, or failure of minimally invasive treatments. At a medium follow-up of 88 months, no local recurrences were verified. Conclusions: osteoblastoma is a rare tumor with difficult diagnosis. Identification is based on symptoms, imaging, and histology. When possible, minimally invasive techniques is preferred for treatment but surgery is still considered the gold standard.

## 1. Introduction

Osteoblastoma (OBL) is a bone-forming tumor accounting for about 1% of all primary bone tumors and 3% of benign bone tumors [1]. Although it is generally benign, some cases of malignant transformation have been described [2].

It usually arises in adolescence and in juvenile ages (second to third decades of life) with a male predominance of 2.5 to 1, although more aggressive forms are described in older patients [3,4].

It involves the spine in about 32% of cases, usually arising in the posterior elements, but it can also develop from the vertebral body, albeit rarely. The other most common sites are the femur (12%), the jaw (11%), tibia (10%), and the foot and ankle (9%) [3,5].

A diagnosis in essential for treatment and is based on symptoms, imaging, and histology.

Sometimes OBL is asymptomatic and is discovered during exams performed for other reasons; when symptomatic, swelling and localized pain are common, but unlike osteoid osteoma, it is less responsive to NSAIDs. Neurological symptoms can also be present in spine sites. Additionally, systemic symptoms such as fever and weight loss can be present [6,7].

Upon imaging, OBL can be very similar to osteoid osteoma: a round or oval radiolucent lesion with well-defined margins and variable sclerosis, which usually increases with patient age [3]. Sometimes, it can break the cortical bone, giving it a more aggressive aspect, and in these cases, differential diagnosis versus osteosarcoma can be difficult.

Histology reveals the mineralized bone trabeculae of mature osteocytes with normal nuclei and abundant cytoplasm, surrounded by a single layer of osteoblasts. The aspect is very similar to that of osteoid osteoma; nevertheless, more multinucleated giant cells can be present [4].

In the past, it was considered of the same class as osteoid osteoma and its size was the characteristic that distinguishes between the two diseases; indeed, a diameter inferior to 2 cm was considered suggestive of osteoid osteoma, while a superior diameter was indicative of OBL [8].

While some authors still sustain that it belongs to the same family as osteoid osteoma, today, it is more frequently considered an independent nosological entity [4,5,9]; although, from a histological point of view, they are not distinguishable.

In the past, the treatment of OO was surgical excision; today, minimally invasive radiological techniques, such as thermoablation and, more recently, high intensity focused ultrasound (HiFu), are considered the gold standard [10,11,12].

For OBL, due to its greater size, surgery still plays an important role, even if thermoablation and Hifu can be considered case by case, especially for small lesions [13,14].

Indeed, Arrigoni et all, in 2018, published a series of 11 patients affected by OBL of the spine who underwent radiofrequency ablation, obtaining total relief in all cases [14]; the same group, two years later, presented six cases of intrarticular OBL successfully treated with Hifu without any complications [13].

The aim of the present paper is to analyze surgical indications based on our experience and on the evidences in the literature.

## 2. Materials and Methods

All patients affected by OBL who underwent surgical excision in a tertiary research hospital in January 2009 and December 2018 were reviewed. Eleven patients, nine males and two females, with an average age of 23 years (range, 9–56 years; median, 21 years) were enrolled in the study (Table 1). All cases were discussed in a multidisciplinary team meeting with orthopedic surgeons, radiologists, oncologists, and radiotherapists to ensure the best possible treatment. 

Diagnoses were performed based on the histologies, reviewed by expert pathologists trained in musculoskeletal tumors, imaging, and symptoms. They were distinguished from OO because, although histologies are similar, OBLs usually present more multinucleated giant cells than OOs; lesions bigger than 2 cm with a no characteristic pain (no nocturnal and no effective response to ASA) were considered OBLs.

Enneking staging was used to quantify the aggressiveness [15]. It distinguishes between three stages: stage 1 is characterized by well-demarcated borders suggestive of a latent disease; in stage 2, borders are less clear, but the lesion is still delimited; stage 3 lesions are very aggressive and destroy the surrounding bone.

All patients were preoperatively studied with X-ray, MRI, and CT scans to better understand the anatomical characteristics of the diseases and to find the best therapeutic approach. In unclear cases, FDG-PET or SPECT were performed.

Surgeries were performed by the same surgical team trained in musculoskeletal oncology; antibiotic prophylaxis was performed with 1 g of cephazolin at three administrations per die and prolonged for three days.

The postoperative rehabilitative protocol was discussed case by case, considering the patient’s specific characteristics. Postoperative X-rays were performed on all patients. X-rays and gadolinium-enhanced MRIs were repeated at 40 days and every 6 months for the first three years and then every year.

The epidemiological aspects, size of the disease and site of onset, symptoms, surgery type, indications, and results are reported for every case.

The study was conducted in accordance with the Declaration of Helsinki Ethical Principles and Good Clinical Practices. All patients provided their consent to use of their data for research purposes and publication.

## 3. Results

The patients’ main characteristics and results are reported in Table 1. 

### 3.1. Epidemiology

The present series was composed of 11 patients, 9 males and 2 females, with an average age of 23 years (range, 9–56 years; median, 21 years) (Table 1). According to Enneking’s system for the Staging of Benign Bone Tumors, four stage 3 and seven stage 2 tumors were reported [15].

### 3.2. Diagnosis

Diagnoses were performed using CT-guided trocar biopsy in 8 out of 11 cases, in which the diagnosis was unclear in 3 cases but a benign nature was assessed; in two cases, the diagnosis was intraoperative; and in the last case, the diagnosis was made during a first unsuccessful surgery performed in a non-specialized center.

### 3.3. Symptoms

The most frequent symptom was pain, complained by all patients; the patient with OBL of the D3 pedicle also presented irradiated pain (case 7).

### 3.4. Surgeries

Intralesional surgeries were performed in 9 out of 11 cases (phenol was used in all cases except for spine localizations); in the two remaining cases, wide resection was performed.

In 6 out of 11 cases, reconstruction was performed: in 3 cases, with homoplastic bone chips (cases 2, 3, 5, and 7) and, in 2 cases, with autoplastic bone harvested from the iliac crest (cases 8 and 9). Reconstruction was not necessary in the other cases.

### 3.5. Clinical Results

All patients reported pain resolution at the first postoperative control about 40 days from surgeries.

### 3.6. Complications

No early or late complications occurred after the surgical procedures.

### 3.7. Histologies

Our pathological findings confirmed the preoperative and intraoperative diagnoses in all cases. The histological examination showed a sharply demarcated lesion composed of irregular interconnecting trabeculae of woven bone rimmed by plump osteoblasts and scattered osteoclasts with intervening loose fibrovascular tissue. Scattered mitoses with no atypical forms were observed.

### 3.8. Indications for Surgery

Surgical removal was indicated in three cases because the lesion was very close to their nerves so thermoablation was judged to be too dangerous (cases 1, 7, and 11). In two cases, the diagnosis was unclear so surgery and an intraoperative histological examination were preferred (cases 2 and 4). In one case, there was a risk of fracture (case 3) so surgical reconstruction was necessary. In one case, the lesion was too large for RF (case 5). In one case, the OBL was near the joint (case 8), and in one case, surgery was performed after RF failure (case 9). In the last two cases, OBL was close to the nerves and the diagnosis was unclear so surgery was highly recommended (cases 6 and 10).

### 3.9. Follow-Up

The medium follow-up was 88 months (range, 28–155 months; median, 80). At the last follow-up, all patients were alive without evidence of local disease.

## 4. Discussion

**Figure 1:**
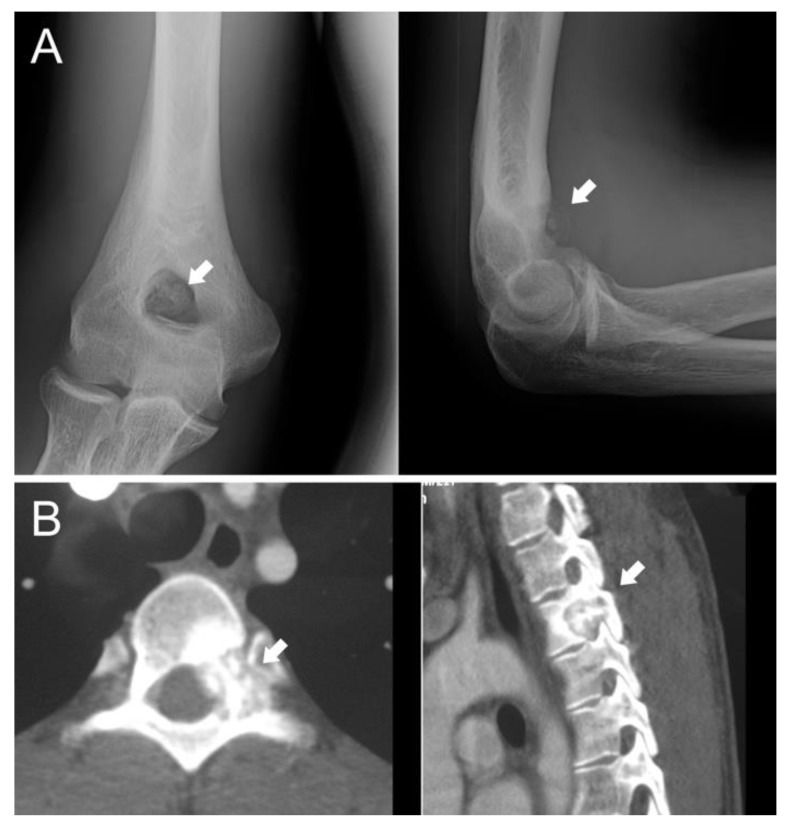
(**A**) Twenty-two-year-old male patient affected by stage 2 distal humerus OBL (white arrows): the lesion was considered suitable for intralesional surgery; (**B**) twenty-one-year-old male patient affected by a D3 left pedicle OBL (white arrows): wide resection was performed to decrease the risk of local recurrence.

The gold-standard treatment for OBL is surgery; generally, intralesional surgery is suggested for grade 2 lesions (Figure 1A), whereas wide resection is more indicated for grade 3, more aggressive tumors or for lesions located in areas where a possible local recurrence could be difficult to treat (Figure 1B). Indeed, the site of onset also has to be assessed; a more aggressive lesion could be treated intralesionally if located in the distal femur, while lesions located in the spine or pelvis are more suitable for resection even if they do not have an aggressive appearance.

Radiotherapy is also considered for the treatment of OBLs located in the spine or sacrum, where wide resection is difficult and the risk of complications and loss of function is consistent [16,17].

In the last decade, the improvement of minimally invasive radiological techniques has introduced valid alternatives that must be considered, moreover for small lesions that are not at risk of fracture and far from neurovascular bundles. 

While in OO, imaging is very characteristic and treatment can be performed without histology, OBL presents a less clear aspect and pain is not as typical as in OO. The treatment has to be based on a diagnosis performed on the histological and clinical aspects: lesions with irregular shape, size bigger than two centimeters, pain not nocturnal and not strictly responding to acetylsalicylic acid, and coherence with a diagnosis of OBL.

In our series, biopsy was performed in all cases, in which three cases did not have clear diagnoses although a benign nature was demonstrated. Before proceeding in the curettage, the intraoperative biopsy confirmed our hypothesis.

**Figure 2:**
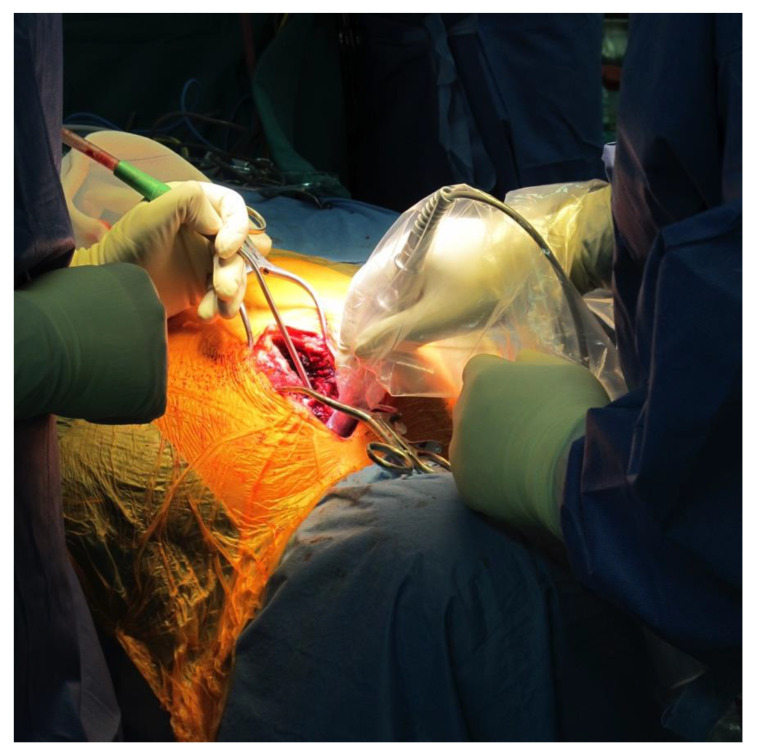
Intraoperative gamma-probe was used to localize the lesion; it can be used to ensure removal of all of the pathologic tissue.

In the presented series, a proximity to nerves was the main indication for surgery; indeed, thermoablation can cause them damage. Additionally, tumor size and shape have to be considered: an irregular shape can be a contraindication for minimally invasive treatment, such as a risk of fracture. In case 3, we preferred to surgically remove the tumor and then to fill the cavity with a homograft to decrease the risk of great trochanter avulsion (Figure 2). Localization should also be considered in the indications for surgery.

In the following paragraphs, OBLs are discussed based on the site of onset starting from the most frequent sites, spine and long bones.

### 4.1. Spine

The most frequent site is the vertebral column (28–36%), mainly the posterior elements and pedicles (Figure 1B) [18]. OBL surgery plays an important role in the spine: the vicinity of the dura and nerve roots limits the application of thermoablation techniques although, with improved expertise, minimally invasive technique are gaining more space also in this complex localization.

The surgical indication is also based on the aggressiveness of the diagnosis: intralesional surgery is suggested for stage 2 OBL, whereas en-bloc resection is more suitable for stage 3. Wide resection decreases the risk of local recurrence, which could be difficult to treat [15,19].

In 2012, Boriani et al. published their experience on 51 cases, clarifying indications and suggesting RT for stage 3 lesions where wide resection is unfeasible or unacceptable for functional sacrifices [19].

A possible problem of spinal OBL is localizing it, moreover, when it is little [20]. Inserting a landmark under CT control before surgery or using a gamma-probe after administration of radioactive bisphosphonates (Figure 2) could be valid suggestions.

Czigléczki et al., analyzing their experience on 31 cases of OBL of the occipito-cervical junction, also suggested preoperative embolization to decrease the risk of bleeding [21].

If the OBL is big and an instability could occur after its removal, stabilization is suggested.

**Figure 3:**
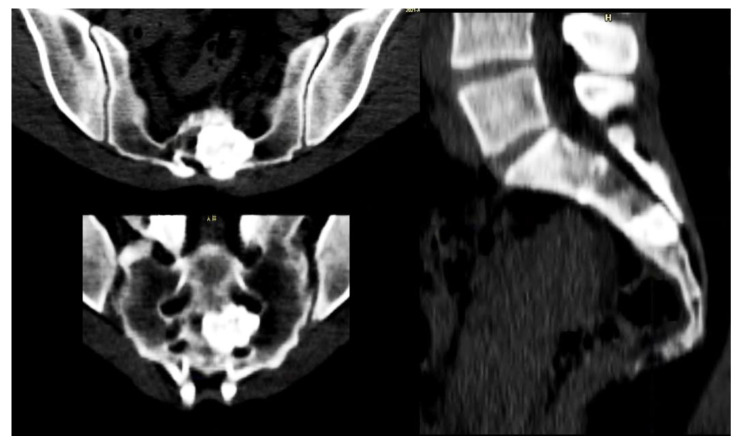
Forty-seven-year-old female patient affected by OBL of S3.

Particular attention has to be reserved for lesions located in the sacrum, where the recurrence rate is higher than in other areas, possibly due to the complex anatomy and the presence of the sacral roots (Figure 3) [22].

Wide resection in large tumors is indicated to decrease the risk of local recurrence, which could be difficult to treat [23].

### 4.2. Long Bones

**Figure 4:**
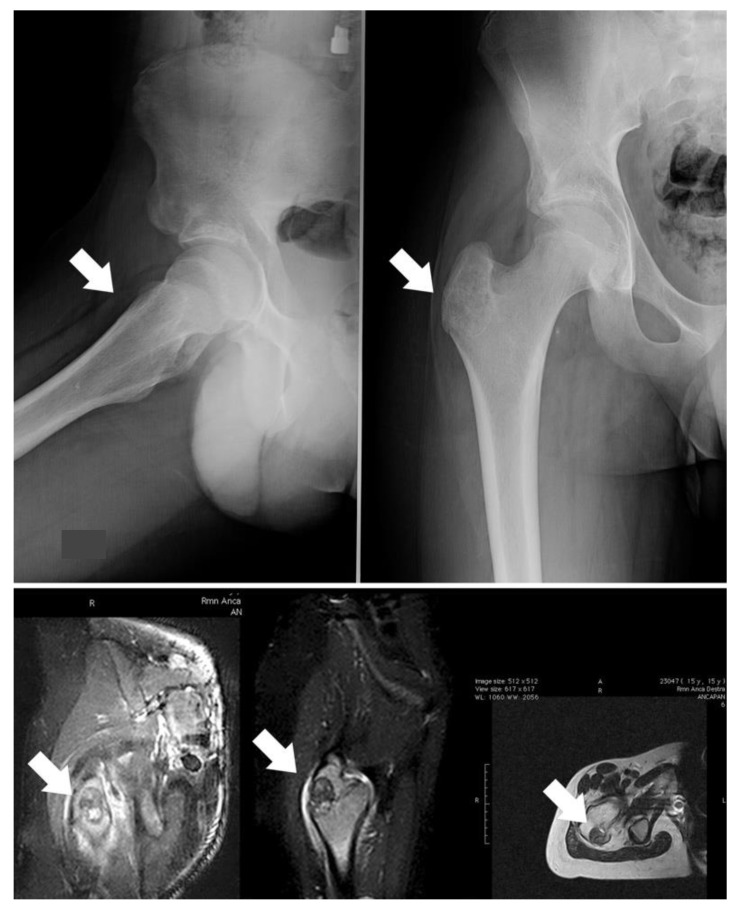
Case 3, a male patient with OBL of the right trochanter; on the upper image, the X-ray shows a quite well-defined osteolytic lesion (white arrows); on the lower image, the related MRI highlights the important oedema surrounding the OBL. The indication for surgery was based on the risk of a fracture due to both eventual RF thermoablation and the action of pelvic-trochanteric muscles.

When OBLs are located in the limbs, an intralesional approach can be considered the best-choice treatment, although, if the lesion is small, thermoablation has to be carefully assessed. Surgery is necessary in cases with a risk of fracture (case 3; Figure 4) because bone filling is required. Resection can also be considered for OBLs located near neurovascular bundles.

Although OBL is common in the limbs, few series of surgically-treated OBLs are available. 

In 2017, Angelini et al. published 13 cases from 1975 to 2012 of OBL located in the elbow (10 cases in the distal humerus, 2 in the proximal ulna, and 1 in the proximal radius), of which 12 were surgically treated [24]. They reported successes in all cases except one that had a local recurrence, treated with a further curettage. 

In 2018, Moreira et al. published a case of OBL onset two and a half years after a clavicle fracture [25]. It was successfully treated with intralesional curettage and allograft packing. Although disease onset after trauma has already been described for osteoid osteoma, this was the first case published in literature of post-traumatic OBL onset.

### 4.3. Hand and Foot

When located in the hand and in the foot, surgical removal can be indicated; indeed, minimally invasive techniques could damage neurovascular bundles and decrease bone segment resistance in the fingers, in the metacarpi, and in the metatarsi; in the carpus and in the tarsus, thermoablation could damage the nearest bones and joints. In the case of an aggressive appearance, wide excision is advisable [26].

Several case reports about OBL of the tarsus are present in the literature. Duan et al. published a case of arthroscopic removal of a lesion located in the talar neck [27]; in 2017, Elsayed et al. published a case of OBL of the talus successfully treated with curettage [28].

### 4.4. Ribs and Sternum

The ribs are involved in about 5% of all cases [3], while the sternum is a very rare localization [29]. No specific series are present in the literature; only case reports are available to evidence this unusual localization [30,31,32]. The gold-standard treatment is based on complete excision, moreover, for big or stage 3 lesions. Minimally invasive radiological techniques are also suggested for small tumors; nevertheless, attention has to be paid to decreasing the risk of lung damage. In 2019, Basappa et al. published the case of a 19-year-old patient affected by rib osteoid osteoma in which an artificial pneumothorax was induced to protect the lung [33].

Although biopsies were not always performed in the literature, our group strictly sustains them. 

### 4.5. Skull

OBLs are localized in the skull in just 2–4% of cases, more frequently in the frontal bone (almost half of the cases) [34]. Surgery is considered the main approach in these cases; wide resection is suggested to decrease the risk of a difficult-to-treat local recurrence [35]. Indeed, Garvayo et al. recently published a systematic review on surgically treated OBLs of the skull, showing that wide resection was performed in 64.4% of cases; that partial/subtotal resection was performed in 19% of cases; and that, in the remaining cases, no information about the degree of surgical resection was reported. The recurrence rate correlated with the resection margin, being 12.5% and 30% in the first and in the second groups, respectively [34]. All recurrences were successfully treated with wide resections.

## 5. Conclusions

OBL is a rare tumor with a difficult diagnosis. Identification is based on symptoms, imaging, and histology. When possible, minimally invasive techniques is preferred for treatment. Surgery is still the first choice in the case of lesions near neurovascular bundles or joints, of those located in areas with a risk of fracture, when reconstruction is necessary, when the diagnosis is unclear, of lesions located at sites difficult to treat in the case of local recurrence such as in the spine or pelvis, and of lesions located in the hand and in the foot.

Further studies with more consistent series are necessary to clarify the presented results.

## Figures and Tables

**Table 1:** Patients’ clinical characteristics.

PT	Sex/Age	Site	How Diagnosis Was Obtained	Major Diam (mm)	Enneking Stage	Symptoms	Surgery Type	Local Adjuvant	Reconstruction	Indications	F.U. (Months)
1	F,15	R S3-S4	CT-trocar biopsy	20	2	pain	i	phenol	none	near nerve roots	28
2	M,9	L-femoral neck	CT-trocar biopsy (unclear)	23	3	pain	i	phenol	hbc	unclear diagnosis	35
3	M,15	R trochanter	CT-trocar biopsy	27	2	pain	i	Liquid nitrogen	hbc	risk of fracture	50
4	M,16	R distal phalanx (first toe)	intraoperative	20	2	pain	i	liquid nitrogen	none	unclear diagnosis	53
5	F,21	L scapula	CT-trocar biopsy	25	2	pain	i	liquid nitrogen	hbc	size	59
6	M,22	R distal humerus	intraoperative (unclear)	28	2	pain	i	phenol	none	unclear diagnosis, near nerves	80
7	M,21	L D3 pedicle	CT-trocar biopsy	21	3	pain, irradiation	w	none	hbc	near nerve roots	116
8	M,56	L proximal ulna	CT-trocar biopsy	25	2	pain	i	phenol	abc	near the joint	120
9	M,21	L acetabulum	CT-trocar biopsy	35	3	pain	i	phenol	abc	failed Radiofrequency thermoablation	148
10	M,36	L distal femur	CT-trocar biopsy (unclear)	21	2	pain	w	none	none	unclear diagnosis, near neurovascular bundle	155
11	M,19	L D12 pedicle	previous surgery	23	3	pain	i	none	none	near nerve roots	120

Diam: diameter, F: female, M: male, R: right, L: left; i: intralesional, w: wide; hbc: homograft bone chips; abc: autoplastic bone chips (iliac crest).

## Data Availability

The data are available only upon request due to privacy and ethical restrictions.
